# Problem-based learning for inter-professional education: evidence from an inter-professional PBL module on palliative care

**Published:** 2013-03-31

**Authors:** Nora McKee, Marcel D’Eon, Krista Trinder

**Affiliations:** University of Saskatchewan, Saskatoon, Saskatchewan, Canada

## Abstract

**Introduction:**

The objective of this article was to analyze the theory and pedagogical basis of the use of problem-based learning (PBL) for inter-professional education (IPE) in undergraduate health science education and present evidence from a palliative care iPBL (inter-professional PBL) module that confirms the importance of the two methodologies being used together.

**Methods:**

More than 1000 student surveys collected over 4 years were analyzed for components of usefulness, enjoyment and facilitator effectiveness. A retrospective self-assessment of learning was used for both content knowledge of palliative care and knowledge of the other professions participating in the module.

**Results:**

Statistically significant gains in knowledge were recorded in both areas assessed. Medical students reported lower gains in knowledge than those in other programs. On a scale of 0 to 6, mean scores were moderately high for usefulness (4.37) and facilitator effectiveness (5.19). Mean scores for enjoyment of the iPBL module were very high at 5.25.

**Conclusion:**

There is strong theoretical and empirical evidence that PBL is a useful method to deliver IPE for palliative care education. With the evidence presented from the palliative care iPBL it is our contention that PBL inter-professional cases should be utilized more often, incorporated into IPE programs generally, and researched more rigorously.

## Introduction

We have been engaged in the practice of inter-professional education (IPE) for over 10 years and have found problem-based learning (PBL) to provide a successful method or platform for the delivery of IPE case discussions. In this article we outline first the theoretical and empirical evidence found in the literature around the question of inter-professional problem-based learning (iPBL) and then add results from a recent study where IPE around palliative care was created through a PBL case experience.

### Theoretical evidence for inter-professional education and problem-based learning

Inter-professional education (IPE) has been supported by various authors for many reasons. IPE may be one part of reforming the management of many complex conditions such as HIV/AIDS.^1^ IPE may also help health professionals to work together effectively by training them to do so in their undergraduate or pre-licensure professional training programs.^2^–^4^ IPE could promote inter-professional competencies such as understanding of professional roles, communication and negotiation skills, enhanced patient/client-centered care, quality improvement,^5^–^8^ and professionalism.^9^ There is considerable hope that IPE will help address many challenges in health care. But how should it best be organized?

Theorists have suggested that IPE should make extensive use of relevant, contextualized, well-structured, and progressively more complex cases through the expert application of cooperative and experiential learning principles.^3^,^8^,^10^–^13^ Furthermore, D’Eon^10^ asserts that inter-professional case discussions and studies must be organized to include the five essential features of cooperative learning as described by Johnson et al^14^: positive interdependence, face-to-face interaction, social skills, group processing, and individual accountability. Similarly, students working in cooperative groups on relevant and realistic problems, cases, or situations should cycle through the four stages of experiential learning: planning, acting, observing, and especially reflecting.^15^ These are not new or unique approaches to a quality educational experience. As both D’Eon^10^ and Freeth^5^ assert, all that we know about good educational practice applies to IPE. This sound advice assumes that before students engage in application and problem-solving they need knowledge and information. This foundational knowledge can be provided through a variety of methods including specifically organized didactic sessions or independent, self-directed research.

Problem-based learning (PBL), distilled to its core, is a variation of a small group case study approach that presents a situation (problem) to learners for which, by design, they are generally unprepared.^16^,^17^ Collectively and individually the learners are therefore required to identify and then seek out the knowledge they can subsequently use to address the case before them. They also learn from one another and are both teachers and learners in this cooperative process. The purpose (as opposed to the process described above) is to help students learn both basic and clinical sciences in the context of patient problems.^16^ Problem-based learning (PBL) incorporates many important principles of cooperative and experiential learning and therefore brings several natural strengths to IPE.^6^,^11^,^18^,^19^

Some researchers have found that PBL fosters a motivational environment that enhances the attainment of disciplinary knowledge and facilitates collegial group work.^19^,^20^ However, others have argued that the effectiveness and sustainability of students’ retained knowledge using a PBL approach compared to conventional curricula have not been sufficiently tested and no firm conclusions can yet be drawn.^21^–^26^ Some systematic reviews on the effectiveness of PBL^27^,^28^ concluded that the existing literature provides inadequate or equivocal evidence about the effectiveness of PBL. A more recent review^29^ found evidence to suggest that PBL enhanced student ability to deal with uncertainty, to understand ethical and legal issues, to communicate effectively, and to sustain life-long learning. Albanese^16^ has become cautiously positive about the effectiveness of PBL based in part on the following recent reports. A study based on 10-years of experience before and with a hybrid PBL model at the University of Missouri-Columbia^30^ found impressive gains on both Step 1 and Step 2 USMLE examinations. Schmidt^31^ used grouped self-assessments by students from PBL and non-PBL medical schools and found large differences especially in interpersonal competencies and self-directed learning but also in general academic competencies. Finally, Schafer^32^ found comparable gains by PBL and non-PBL students on basic science learning but by the beginning of the fifth semester the PBL students surpassed the non-PBL students on clinical reasoning by effect sizes greater than *d* = 1.17. PBL has theoretical strengths and now it seems that the positive empirical evidence is beginning to accumulate. More theory-driven research is needed to establish and measure the mechanisms by which PBL seems to work.^23^

### Empirical evidence for iPBL from previous studies

There have been only a few articles and evaluations of iPBL in the literature. All of them report some success at using PBL as a delivery method for IPE. Some involve only two or three professions^7^,^33^–^36^ and sometimes only volunteers.^7^,^35^,^36^ More recently, D’Eon et al^18^ described the evaluation of an iPBL module on caring for persons with HIV/AIDS involving up to 300 students from seven different health and human science programs for many of whom this was a mandatory curricular experience. That iPBL has been used so little is surprising given the obvious connection between the educational requirements for IPE and the strengths of PBL.

Thompson^37^ reviewed the literature available regarding the theoretical use of IPE and PBL together. She concluded that there is favourable evidence for improving attitudes towards other professional groups by incorporating the two concepts into curricula. She found no evidence confirming skill and knowledge acquisitions and recognized that these competencies are difficult to measure. Barr^6^ specifically mentions PBL as one promising method among many, while Dahlgren^11^ suggests that PBL is an excellent match for IPE. Freeth^5^ on the other hand advises the use of other delivery methods over PBL. She proposes instead various case-based or problem-oriented approaches (consistent with D’Eon^10^), other higher fidelity simulations, clinical shadowing, and inter-professional clinical student placements. Though there is some controversy about using PBL for IPE there seems to be much theoretical and growing empirical evidence for using iPBL.

The following report of an evaluation done on a Palliative Care Inter-professional PBL module is meant to add to this body of previously published empirical evidence. The information has additional weight because it includes data from a large number of students, involved in a compulsory educational activity, over several years and involves a number of different professional programs.

### Palliative care iPBL at the University of Saskatchewan

The University of Saskatchewan has a generally conventional curriculum for its health science students with large-group interactive lectures, small-group case discussions, and clinical skills instruction. In 2001, the School of Physical Therapy attracted by the potential for learning through PBL developed and offered their students a uni-professional PBL module in the care of persons with HIV/AIDS with the intent to make the experience inter-professional. In the subsequent years, students from medicine, pharmacy, nutrition, nursing, social work and clinical psychology were added, creating a true iPBL experience.^18^ This module was successful with high student satisfaction scores and statistically significant knowledge gains measured by self-evaluation in both the content area of HIV/AIDS and in the knowledge of the other participating professions. Pre- and post-tests were also used to confirm gains in learning. Tutors rated the module a positive experience and reported that they learned as well.

Palliative care education for undergraduate health science students seems to be in some disarray: fragmented, poorly assessed and often uni-professional.^38^ Different educational models and programs have been proposed to attempt to meet this challenge.^39^–^42^ Many of those implemented have been clinical in nature and often with in-hospital teams but are often small-scale, voluntary and inconsistent. Frequently the methods chosen for learning about palliative care were case-based, but not always offered in an inter-professional setting. Since palliative care is inter-professional by nature, it only seems logical, as Freeth^5^ and Wee et al^43^ suggest, that undergraduate students should learn palliative care in an inter-professional setting. iPBL is therefore recognized as a good instructional method for delivering palliative care education.

In 2006 educators from medicine, nursing, and pharmacy at the U of S collaborated to create and pilot an iPBL palliative care case, the evaluation of which was described by McKee et al.^36^ Because of the successes of the HIV/AIDS iPBL module and the Palliative Care iPBL pilot, the Palliative Care case was implemented on a large scale to multiple programs at the University of Saskatchewan in 2007 and has continued through to the present time (see Table 1). Due to scheduling conflicts program participants varied from year to year and in 2010 the entire case was reduced to just two afternoon sessions rather than three. Despite these small variations and other minor case modifications and enhancements, all four years running the Palliative Care iPBL module involved approximately the same size and composition of student groups and essentially the same clinical end-of-life case. Students were sorted into groups of 8–12 members from a variety of professions to progress through the case of a gentleman’s end-of-life care both at home and in hospital, and ended with his death in the final session (see Appendix 1 for an example of one page of the case used). Traditional PBL approaches were used such as establishing ground rules, identifying and reporting issues known, and researching unknown concepts or ideas.

As iPBL was a new instructional method at our facility we wanted to ensure that tutors were well trained.^44^ Tutors were assigned to each group and all were prepared by attending a two, half-day tutor-training workshop. It included pre-workshop preparation, small and large group discussions, observations of a “real” PBL group interaction and role play practice. University and community faculty were recruited by each program. Since tutors were not content experts and were often from different programs than the students in their groups, some general information about palliative care was provided in advance by email. A tutor orientation and review of documentation was held the first day of the module and during the module, support was offered to facilitators by informal pre- and post-session “coffee” gatherings, led by one of the senior tutors.

## Methods

Evaluation of our Palliative Care iPBL module was approved by the Research Ethics Board (Behavioural) of the University of Saskatchewan. The focus of the evaluation was to determine (1) student satisfaction and (2) learning about palliative care and the role of their inter-professional colleagues.

Students that participated in the evaluation were from health professional programs: medicine, nursing, pharmacy, nutrition, social work, physical therapy and one individual from clinical psychology. A previously developed and tested student questionnaire^18^ with Likert-style responses and space for additional descriptive comments was given to each student at the conclusion of the iPBL module. A principal components factor analysis that was performed on the student satisfaction survey yielded three categories: *usefulness, enjoyment,* and *facilitator effectiveness*. Response options for all items ranged from 0 (strongly disagree) through 6 (strongly agree), with 3 indicating “don’t know” and an additional option to indicate “not applicable”. *Usefulness* consisted of seven survey-items related to motivation, rewards, relevance, and worth of various aspects of the iPBL module. This factor was found to be internally consistent with a reliability coefficient of α = .73. The *enjoyable* factor was composed of five items and addressed student enjoyment of the iPBL module. This factor was also found to be internally consistent (α = .85). *Facilitator effectiveness* was the combination of only two items that asked about facilitator skill in guiding the group process. This factor possessed a high degree of internal consistency (α = .95). We asked about facilitator effectiveness because this is a critical component of the PBL learning experience and we wanted to know if we needed to train our tutors better or differently.

The questionnaire also included a retrospective self-assessment of learning in both the content area of palliative care and knowledge of the other professions participating. Following are the self-assessment questions asked of students:

Consider the extent of your CURRENT knowledge of Palliative Care process and services available in Saskatoon to be 9 out of 9. Using a number between 0 and 9, indicate what your knowledge level was before beginning this inter-professional PBL module (where 9 would indicate that you know everything already and a 0 would mean that you did not know anything before).

Consider the extent of your CURRENT knowledge of what other disciplines can bring to the care of Palliative Care patients/clients to be 9 out of 9. Using a number between 0 and 9, indicate what your knowledge level was before beginning this inter-professional PBL module.

Self-assessment responses were scored on a 10-point scale, with higher scores reflecting greater gains in knowledge. Students were also provided with space to provide comments about the session.

### Analyses

Univariate ANOVAs with post-hoc Tukey tests were conducted to compare differences between years and programs. Independent-samples t-tests and effect sizes (Cohen’s *d*) were calculated for the retrospective pre- and post self-assessments. Cohen’s *d* is a standardized measure of the difference between two means, where *d* = .2 indicates a small effect size, *d* = .5 indicates a medium effect size, and *d* = .8 is considered a large effect size.

## Results

In this section we report on the students’ experience and their perceived gains in knowledge. Data analyzed were based on an 85% response rate over the 4 years.

### Knowledge of Palliative Care

Mean perceived gains in knowledge about the palliative care process and services are reported in Table 2. Based on the retrospective self-assessments, there was a statistically significant gain in knowledge of palliative care overall for all years. Furthermore, effect sizes, as measured through Cohen’s *d*, were large for all years. There were several statistically significant differences among professions overall for all years based on the self-assessments (see Tables 3a and 3b).

Specifically, medical students reported lower gains in knowledge than those in pharmacy, nutrition, and social work. Physical therapy and pharmacy students reported significantly greater gains than students in many of the other programs.

### Knowledge of other professions

Gains in knowledge about other professions were statistically significant in all years with very large effect sizes from *d* = 2.67 to 3.76 (Table 2). Overall, medical students reported gains in knowledge that were significantly lower than those reported by students in physical therapy, pharmacy, nutrition, and social work (Table 3b).

### Student satisfaction

#### Usefulness

Over the years student responses indicated that they found the Palliative Care iPBL module to be moderately useful with an overall mean of 4.36 (on a scale of 0 to 6). The session provided in the summer of 2009 was considered significantly less useful than those offered in 2007 and 2009 (Table 4a). As well, the session provided in the summer of 2010 was rated as significantly less useful than the sessions provided in 2007, 2008, 2009, and 2010 (see Table 4b). Both physical therapy and medical students rated this iPBL module as significantly less useful than students in pharmacy, nutrition, nursing, and social work (see Table 3). Student comments reflecting the usefulness of iPBL session included:

“One of the most relevant and applicable things thus far in NEPS [nursing program]. Something that we can use and grow from.”

The group worked rather well together, it really gave me confidence in my role as a social worker as part of an interdisciplinary team.

The majority of students found the iPBL modules useful. However, some students commented that this module was not representative of what they might encounter in a work setting, as reflected in the following quotes:

This would be more effective if we met in a clinical setting, hands on with a real patient. Overall, great experience.

I think the way these PBL are set up doesn’t actually reflect inter-professional in the real world. To come in, make objectives and to then all leave and research on our own is not inter-professional to me.

Several students who attended the summer iPBL sessions commented that the session would have benefitted from the inclusion of additional professions. Furthermore, physical therapy students did not feel that this session was very relevant to their profession. This supports the lower usefulness ratings given by students in these sessions and is reflected in the following comments:

“Have more colleges involved. The main purpose is to work as an interdisciplinary team and having more than four colleges would be beneficial.”

“I felt the group worked well together. I did feel though this PBL was geared much more toward nursing and I felt there could have been more for MPT (Masters of Physiotherapy) students.”

#### Enjoyment

Students rated the modules as highly enjoyable with a total mean score of 5.19 out of 6.0. Students attending the summer sessions generally rated the iPBL module significantly less enjoyable than most of the regular winter sessions (Table 4). Comments reflecting student enjoyment included:

“This experience was far beyond any expectations I had and was very enjoyable and helpful.”

“I enjoyed it and really helped me to learn about teams like this and what is possible out there.”

#### Facilitator Effectiveness

Students perceived the facilitation to be very effective (total mean score of 5.19 out of 6.0). Student comments reflecting satisfaction with facilitation include:

“Facilitator was excellent and was very helpful in guiding the process and encouraging us to find info on our own.”

“Facilitator was perfect. Knowledgeable enough on the subject to give good cues and prompts to direct the process and keep it going in the right direction.”

Although a majority of students were satisfied with their facilitator, dissatisfaction with facilitation reflected the desire for either more or less guidance from the facilitator. This is illustrated in the following quotes:

“Probing questions should not be asked by Facilitator when the group has already answered the question.”

“PBL group leader was slightly over the top and scrutinized too much detail.”

## Discussion

This iPBL module on palliative care was very highly rated by the students and showed large knowledge gains using grouped self-assessments. These results are in fact similar to those obtained in the HIV/AIDS iPBL module reported by D’Eon et al.^18^ The ratings for usefulness and enjoyment were high across programs. It is particularly surprising that, over 4 years as a compulsory learning activity involving a wide range of programs, the students consistently enjoy the module enough to rate it as greater than a mean of 5 out of a maximum score of 6. Being entertained *is not* a goal of the project but a strong element of enjoyment suggests the students have a positive attitude towards learning about inter-professional collaboration and palliative care, or the process or both -- which definitely *is* a goal of the project, and potentially contributes to the attainment of other goals.

The self-assessments, however promising as predictors of criterion measures, may be less accurate than more objective measures of knowledge. D’Eon et al.^45^ and Blanch-Hartigan^46^ both concluded that self-assessments are good proxies for criterion measures though there is some controversy over this approach.^47^,^48^ Self-assessments were used effectively by Ponzer et al.^49^ and Hallin et al.^50^ to demonstrate increased collaborative and professional competence in clinical settings. A validated measure of palliative care knowledge and professional roles ought to be used in subsequent studies to better determine knowledge gain and to further validate the grouped self-assessments.

Medical students reported learning less about palliative care and about their colleagues than students from other professional programs. One can only speculate as to the reason for this. Medical students have potentially spent more time at university, often having obtained a degree before entering medical school. A few in fact had been health care providers in other professions before entering medical school. Medical students in this iPBL module were in their second year of study in all years of the module, so may have already had some contact with care at the end of life in their early clinical experiences. Though the actual differences are relatively small it is an important consideration. The case needs to be written and participating programs selected such that the knowledge and skills are at an appropriate level and relevant to the future careers of the students.

It is worth commenting on the two summer sessions which were rated significantly lower in both categories of usefulness and enjoyment. Due to scheduling issues, the physical therapy and nursing students were unable to participate in the regular palliative care iPBL during the winter. Since their program leads did not want them to miss the experience, they coordinated a module in June with just these two programs. The lower ratings may be a product of fatigue at the end of the academic year and physical therapy student comments indicated that some do not see palliative care as a prominent part of their future careers. Some of the other comments suggested they were less engaged because they felt their groups were missing some of the key professionals.

Many student concerns about the iPBL module are valid. It would be a better experience if they were in a real clinical setting dealing with real patients and real health care providers. Even including a patient or family caregiver in each group would heighten the reality. Many PBL sessions at other health sciences schools have been enhanced through video, role play, and virtual patients.^51^–^53^ These enhancements would add to the quality of the experience. Some students are frustrated by the PBL process and lack of final “answers” but this occurs in uni-professional PBLs as well. Ongoing attention needs to be paid to process evaluations and student concerns.

Additional research is required to test the efficacy of the iPBL approach in relation to other comparable interventions such as case discussions, simulations, and clinical placements. One such educational approach would be large-group interactive sessions followed by discussions of the same case in inter-professional groups. This would isolate the more self-directed and independent research features of problem-based learning. It would be possible, even with large numbers of participating students, to randomly assign half to the iPBL condition and half to the large group/case-based discussion condition. Alternatively, a cohort study design could also be used, though not as powerful as a randomized controlled trial (RCT), where instead of the iPBL module a large-group/case-based discussion approach would be used for the entire cohort one year. The outcomes from the two years could then be compared. Similarly the value of the inter-professional feature of iPBL could be tested by creating two conditions – one inter-professional and the other uni-professional – for the RCT or the cohort quasi-experiment described above. Using appropriate pre- and post-tests in a case or simulated practice setting^54^ would help establish the relative strength of iPBL compared to alternative approaches and demonstrate the value of independent self-directed learning and inter-professional groups.

The Cochrane review of 2008^55^ encourages studies on inter-professional methodologies to prove the effects on professional practice and health care outcomes. Designing studies to demonstrate an improvement in patient-centered care is the ultimate challenge, one not addressed by this study. Recent work by Hallin^56^ begins that process by creating clinical education wards that are inter-professional and using patient questionnaires to determine if care has been improved by involving teams of students. Patients’ perceptions that communication and collaboration were of higher quality encourages us to continue to find evidence that creating these inter-professional learning environments is crucial.

A particular strength of inter-professional PBL (iPBL) for IPE compared to case-based learning is its relative ease to incorporate into multiple independent curricula. As mentioned earlier, by design all students enter the PBL process generally unprepared but they learn from their own explorations and investigations between sessions and from each other.^17^ It is not necessary, as it is for case-based discussions, that all students have been taught the concepts and principles needed to successfully contend with the problematic aspects of an iPBL case. iPBL eliminates the need for complex curricular coordination of content knowledge and skills because the students respectfully teach each other and themselves much of what they need to know for the case at hand. This makes the self-directed and cooperative learning components of PBL particularly well suited to the logistical challenges of IPE.

### Conclusions

When considering the theoretical and empirical evidence reviewed, IPE and PBL work well together. They create fundamentally positive experiences for health-science students likely because they are experiential, cooperative and case-based. The results from our palliative care iPBL add to the previous evidence and together they support our conclusion.

We assert that PBL is one credible approach to inter-professional education. We base our recommendation on the theoretical connections between IPE and PBL as outlined above, the empirical evidence from previous studies, and our own extensive experience with an iPBL module in palliative care. We are not suggesting that only PBL should be used to deliver IPE or that PBL would be the best way to implement IPE. We merely state, yet contrary to Freeth,^5^ that iPBL is a promising approach that deserves careful scrutiny and cautious experimentation within a program of IPE. It is our contention that use of PBL inter-professional cases be utilized more often, incorporated into IPE programs generally, and researched more rigorously.

## Figures and Tables

**Table 1:** Module participants who completed surveys (2007–2010)

Year (Fall)	Program	# of Students	Level of Students
**2007**	Physical Therapy	28	Year 2 (Bachelor)*
	Medicine	51	Year 2
	Pharmacy	57	Year 3
	Nutrition	24	Year 3

**2008**	Medicine	59	Year 2
	Pharmacy	81	Year 3
	Nutrition	26	Year 3
	Social Work	50	Year 4
	Clinical Psychology	1	Year 3 (PhD program)

**2009**	Medicine	29	Year 2
	Pharmacy	63	Year 3
	Nutrition	15	Year 3
	Nursing	81	Year 2
	Social Work	24	Year 4

**2009 Summer**	Physical Therapy	39	Year 2 (Masters)*
	Nursing	52	Year 2 post-RN

**2010**	Medicine	73	Year 2
	Pharmacy	74	Year 3
	Nutrition	23	Year 3

**2010 Summer**	Physical Therapy	39	Year 2 (Masters)*
	Nursing	41	Year 2 post-RN

* Physical therapy program changed from bachelors to master’s degree in 2009.

**Table 2:** Student self-assessments*

	2007 *M (SD)*	2008 *M (SD)*	2009 *M (SD)*	2009 Summer *M (SD)*	2010 *M (SD)*	2010 Summer *M (SD)*
Gain in knowledge of Palliative Care	6.13 (2.12)	5.38 (2.13)	5.39 (2.01)	4.85 (1.88)	5.37 (2.10)	4.69 (1.84)
Comparison of post and retrospective self-assessments	*t*(159) = 36.59	*t(198) = 35.63*	*t(207) = 38.66*	*t(90) = 24.60*	*t(163) = 32.74*	*t(76) = 22.31*
	*p* < .001	*p < .001*	*p < .001*	*p < .001*	*p < .001*	*p < .001*
	*d = 2.58*	*d = 3.57*	*d = 3.79*	*d = 3.65*	*d = 3.61*	*d = 3.31*
Gain in knowledge of other professions	4.78 (2.19)	4.31 (2.08)	4.46 (1.83)	4.41 (1.71)	3.97 (2.10)	3.68 (2.00)
Comparison of post and retrospective self-assessments	*t(158) = 27.57*	*t(198) = 9.17*	*t(198) = 34.34*	*t(87) = 24.24*	*t(162) = 24.12*	*t(74) = 15.93*
	*p < .001*	*p < .001*	*p < .001*	*p < .001*	*p < .001*	*p < .001*
	*d = 3.37*	*d = 2.92*	*d = 3.45*	*d = 3.65*	*d = 2.67*	*d = 3.76*

* 0–6 point scale, with higher scores reflecting greater gains

**Table 3a:** Comparing satisfaction and knowledge by program (0–6 scale with higher scores reflecting greater satisfaction)

Program (total number of students)	Usefulness	Enjoyment	Facilitator Effectiveness	Gain in Knowledge of Palliative Care	Gain in Knowledge of Other Professions
	*M (SD)*	*M (SD)*	*M (SD)*	*M (SD)*	*M (SD)*
Physical Therapy (106)	3.94 (0.85)	5.07 (0.68)	4.77 (1.22)	5.53 (1.89)	4.39 (1.94)
Medicine (212)	4.19 (0.86)	5.28 (0.75)	5.16 (1.08)	4.60 (1.90)	3.72 (2.02)
Pharmacy (275)	4.46 (0.68)	5.31 (0.61)	5.35 (0.82)	6.06 (1.96)	4.71 (2.00)
Nutrition (88)	4.60 (0.61)	5.26 (0.61)	5.16 (1.09)	6.82 (2.02)	4.46 (2.31)
Nursing (174 )	4.46 (0.79)	5.24 (0.69)	5.20 (0.89)	4.63 (1.93)	4.17 (1.81)
Social Work (74)	4.64 (0.71)	5.27 (0.72)	5.33 (0.99)	5.33 (2.21)	4.75 (2.00)
Total	4.37 (0.79)	5.25 (0.68)	5.19 (1.00)	5.41 (2.08)	4.33 (2.03)

**Table 3b:** ANOVA results comparing satisfaction and knowledge by program

Category	ANOVA	*Post Hoc*		*Sig.*
Usefulness	*F*(5, 901) = 13.51, *p* <.001	Physical Therapy	– Pharmacy	*p < .001*
			– Nutrition	*p < .001*
			– Nursing	*p < .001*
			– Social Work	*p < .001*
		Medicine	– Pharmacy	*p < .001*
			– Nutrition	*p < .001*
			– Nursing	*p < .01*
			– Social Work	*p < .001*
Facilitator Effectiveness	*F*(5, 897) = 5.45, *p* < .001	Physical Therapy	– Medicine	*p < .05*
			– Pharmacy	*p < .001*
			– Nutrition	*ns*
			– Nursing	*p < .01*
			– Social Work	*p < .01*
Gain in Knowledge of Palliative Care	*F(5,886) = 27.24, p < .001*	Physical Therapy	– Medicine	*p < .001*
			– Nutrition	*p < .001*
			– Nursing	*p < .01*
		Medicine	– Pharmacy	*p < .001*
			– Nutrition	*p < .001*
			– Social Work	*ns*
		Pharmacy	– Nutrition	*p < .05*
			– Nursing	*p < .001*
			– Social Work	*ns*
		Nutrition	– Nursing	*p < .001*
			– Social Work	*p < .001*
Gain in Knowledge of Other Professions	*F(5,871) = 6.46, p < .001*	Medicine	– Physical Therapy	*ns*
			– Pharmacy	*p < .001*
			– Nutrition	*ns*
			– Social Work	*p < .01*

Note: The non-significant ANOVA and post-hoc results were removed for the “Enjoyment” category. ns = not significant.

**Table 4a:** Student satisfaction: Usefulness, Enjoyment, and Facilitator effectiveness*

Year	Usefulness M (SD)	Enjoyment M (SD)	Facilitator Effectiveness* M (SD)
**2007** (Physical Therapy, Medicine, Pharmacy, Nutrition)	4.51 (0.54)	5.35 (0.61)	5.19 (0.86)
**2008** ( Med., Pharmacy, Nutrition, Nursing, Social Work, Clinical Psychology)	4.32 (0.83)	5.27 (0.54)	5.22 (1.11)
**2009** (Pharm., Nutrition, Nursing, Social Work)	4.59 (0.66)	5.25 (0.66)	5.27 (0.87)
**2009 Summer** (Physical Therapy, Nursing)	4.09 (0.82)	5.09 (0.57)	4.79 (1.13)
**2010** (Med, Pharmacy, Nutrition)	4.35 (0.85)	5.37 (0.72)	5.35 (0.97)
**2010 Summer** (Physical Therapy, Nursing)	3.88 (0.93)	4.98 (0.85)	5.03 (1.11)
**Total**	4.36 (0.79)	5.26 (0.68)	5.19 (1.00)

* 0–6 point scale with higher scores reflecting greater satisfaction

**Table 4b:** ANOVA results comparing student satisfaction by year

Category	ANOVA	*Post Hoc*		*Sig.*
Usefulness	*F(914) = 14.07, p < .001*	2009	– 2008	*p < .01*
			– 2010	*p < .05*
		2009 Summer	– 2007	*p < .001*
			– 2009	*p < .001*
		2010 Summer	– 2007	*p < .001*
			– 2008	*p < .001*
			– 2009	*p < .001*
			– 2010	*p < .001*
Enjoyment	*F*(914) = 5.50, *p* < .001	2009 Summer	– 2007	*p < .05*
			– 2010	*p < .05*
		2010 Summer	– 2007	*p < .001*
			– 2008	*p < .05*
			– 2009	*p < .05*
			– 2010	*p < .001*
Facilitator Effectiveness	*F*(910) = 4.68, *p* < .001	2009 Summer	– 2007	*p = .022*
			– 2008	*p = .006*
			– 2009	*p = .002*
			– 2010	*p = .001*

## Appendix 1 Sample page from Palliative Care iPBL module

### Day 1 -- Page 3

After Mr. Semple’s initial assessment by the Palliative Care Nurse Coordinator, some changes were made in his care. The nurse sat down with Mr. and Mrs. Semple again, and discussed additional services available in the health region. They had an open discussion about Mr. Semple’s disease progression and what he was currently hoping for. He agreed to accept palliative services, in particular to have the nurse visit him regularly at home to assess symptoms and also to receive Palliative Care Drug coverage. Referrals were made to outpatient physiotherapy, occupational therapy and the dietitian.

Mr. Semple wished to have a full resuscitation if necessary but promised to look over some information on advance directive planning. Grace asked if someone from spiritual care (preferably Protestant) could possibly visit. Grace shares with the nurse in private, that 4 years ago Mr. Semple and his daughter Sharon had an argument over a relationship she was in. The two of them have only communicated through Grace, since that time.

The nurse coordinator and Dr. Roberts made the following medication changes:

Tylenol #3 was discontinued and he was started on MS Contin 30 mg po bid
Morphine IR 5 mg was started q1h prn for breakthrough pain
Metoclopramide 10 mg po qid was started, taken regularly
Senokot S 2 tabs bid, taken regularly
Ibuprofen 400 mg po qid was added

At his appointment with the radiation oncologist, external beam radiation was used to treat 3 different bony areas.

**You are the palliative care team.**

**What further services could you/will you offer Mr. Semple:**
